# Monitoring Intracellular Calcium Ion Dynamics in Hair Cell Populations with Fluo-4 AM

**DOI:** 10.1371/journal.pone.0051874

**Published:** 2012-12-17

**Authors:** Kateri J. Spinelli, Peter G. Gillespie

**Affiliations:** Oregon Hearing Research Center and Vollum Institute, Oregon Health & Science University, Portland, Oregon, United States of America; Tel-Aviv University, Israel

## Abstract

We optimized Fluo-4 AM loading of chicken cochlea to report hair-bundle Ca^2+^ signals in populations of hair cells. The bundle Ca^2+^ signal reported the physiological state of the bundle and cell; extruding cells had very high bundle Fluo-4 fluorescence, cells with intact bundles and tip links had intermediate fluorescence, and damaged cells with broken tip links had low fluorescence. Moreover, Fluo-4 fluorescence in the bundle correlated with Ca^2+^ entry through transduction channels; mechanically activating transduction channels increased the Fluo-4 signal, while breaking tip links with Ca^2+^ chelators or blocking Ca^2+^ entry through transduction channels each caused bundle and cell-body Fluo-4 fluorescence to decrease. These results show that when tip links break, bundle and soma Ca^2+^ decrease, which could serve to stimulate the hair cell’s tip-link regeneration process. Measurement of bundle Ca^2+^ with Fluo-4 AM is therefore a simple method for assessing mechanotransduction in hair cells and permits an increased understanding of the interplay of tip links, transduction channels, and Ca^2+^ signaling in the hair cell.

## Introduction

Hair cells of the inner ear are specialized sensory cells that rely on intracellular Ca^2+^ for essential cellular functions. In addition to the typical roles that Ca^2+^ plays in other neuronal cell types, such as modulating neurotransmitter release and synaptic transmission [1], Ca^2+^ also influences three components of mechanotransduction. First, Ca^2+^ sets the resting open probability of the mechanotransduction channel [2]; second, Ca^2+^ modulates slow adaptation by changing the activity of the adaptation motor [3]; and finally, Ca^2+^ controls fast adaptation, perhaps by binding directly to the mechanotransduction channel [4]. In addition, intracellular Ca^2+^ may influence the regeneration of broken tip links [5], transmembrane proteins that gate the transduction channel [6]. The transduction channel is Ca^2+^-permeable [7], providing a local source of Ca^2+^ in the mechanically sensitive hair bundle that may modulate these processes; indeed, in one model, the predicted–but never before measured–decrease in resting bundle Ca^2+^ after tip links break is the signal that triggers tip-link regeneration [5].

Typical hair-cell Ca^2+^ imaging experiments use a whole-cell recording electrode to deliver a fluorescent Ca^2+^ indicator [8], [9]. This technique powerfully pairs electrophysiological recordings with accurate measurements of intracellular Ca^2+^ dynamics. However, only one cell is examined at a time, and surrounding hair cells are damaged to gain access to the cell of interest. Because one must individually dialyze each cell, studying a population of cells is time-consuming. Furthermore, the intracellular environment may become compromised during whole-cell recordings; for example, Ca^2+^ buffering by the pipette solution may not replicate that of the unperturbed hair cell.

A handful of previous studies have reported the use of cell-permeable Ca^2+^ indicators in hair cells. To examine defects in the hair-bundle Ca^2+^ pump, Bortolozzi and colleagues used the cell-permeable Ca^2+^ indicator Fluo-4 AM to monitor clearance rates in the bundle after uncaging intracellular Ca^2+^ [10], [11]. Another group loaded hair cells with another cell-permeable Ca^2+^ dye, Oregon Green BAPTA 488 AM, in a semi-intact mouse cochlear preparation, reporting modest changes in cell body fluorescence in response to stimulation of the stapes as an indicator of mechanotransduction [12]. Neither of these studies specifically compared bundle fluorescence to the morphological state of the bundle, presence of tip links, or transduction. One study even showed that imaging hair cells loaded with Fura-2 AM induced death of outer hair cells, presumably due to phototoxicity and Ca^2+^ loading of the cells [13]. Other reports indicated that isolated cells loaded with Fluo-3 AM or Fluo-4 AM showed fluorescence at rest in the cell body but not in the bundle, which likely resulted from damage to tip links and subsequent transduction channel closure [14], [15].

To examine intracellular Ca^2+^ simultaneously in a population of hair cells, we have optimized loading of hair cells with the cell-permeable Ca^2+^ indicator Fluo-4 AM. This minimally-invasive protocol labeled most hair cells in the chicken cochlea while maintaining excellent tissue and hair-bundle morphology. By combining live-cell imaging with scanning electron microscopy (SEM), we correlated Fluo-4 bundle fluorescence with the presence of tip links. Direct displacement of the bundle by a fluid jet verified that the Fluo-4 bundle signal was due to Ca^2+^ entry through functional transduction channels. Both breaking tip links and blocking the transduction channel lowered the intracellular Fluo-4 fluorescence, which confirms that when tip links break, bundle Ca^2+^ decreases.

## Results

### Loading Chicken Cochlear Hair Cells with Fluo-4 AM

Fluo-4 is a high-affinity Ca^2+^ dye that shows a >100-fold increase in fluorescence when bound to Ca^2+^ [16]. With a K_d_ of 350 nM, similar to the buffering capacity of BAPTA (136 nM), Fluo-4 is sensitive enough to detect and report low concentrations of free intracellular Ca^2+^. Modification of the dye with an acetoxymethyl (AM) ester moiety allows this dye to cross the cell membrane, whereupon endogenous esterases cleave the AM group to liberate the active dye. Fluo-4 AM is virtually non-fluorescent, ensuring that the indicator fluoresces only after the AM group is cleaved and Ca^2+^ binds Fluo-4 intracellularly.

**Figure 1:**
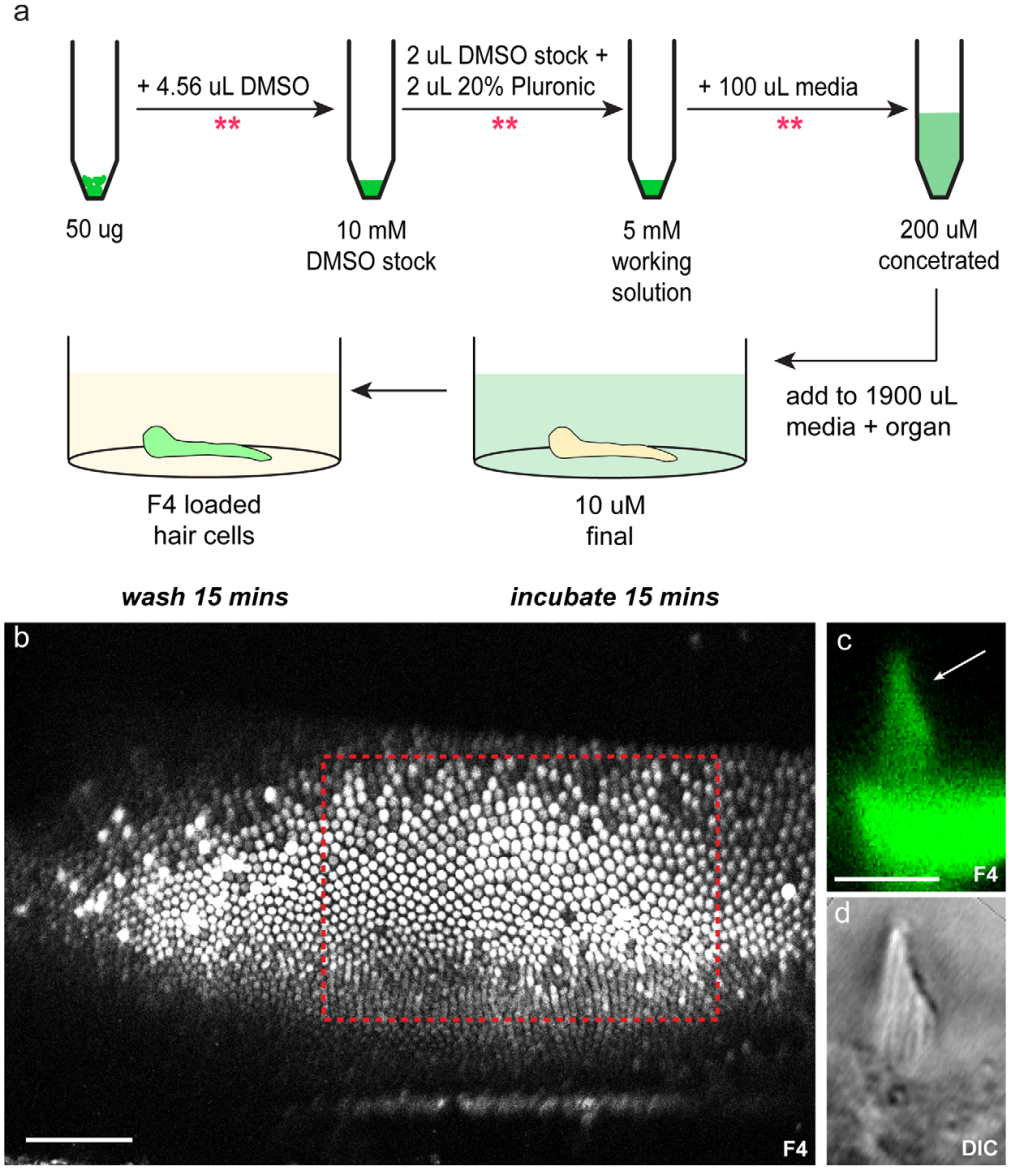
Fluo-4 imaging of cochlear hair cells. (a) Fluo-4 AM dye preparation and loading protocol. Between each of the steps highlighted with pink stars, the dye solution was rigorously vortexed for 1 minute, bath sonicated for 1 minute, and centrifuged. (b–d) Confocal imaging of cochlear hair cells loaded with Fluo-4 AM Ca^2+^ indicator. (b) Low-magnification view of the cochlear epithelium shows that hair cells selectively load with Fluo-4 AM dye. The red box indicates the mid-apical region of the epithelium, which was used for all experiments. (c–d) Side view of a single hair bundle loaded with Fluo-4 (c), with corresponding DIC image (d). Arrow in (c) indicates a tip blush of Ca^2+^ entering through mechanotransduction channels. Scale bar in b is 100 µm and scale bar in c is 5 µm.

To load hair cells with Fluo-4 AM, post-hatch chicken cochleae (P0) were removed from the skull and acutely dissected at room temperature, with removal of the tectorial membrane after incubation with the protease subtilisin. Careful preparation of the Fluo-4 AM dye, as well as optimization of incubation time and temperature, were necessary to ensure the dye stayed in solution and was taken up by hair cells with minimum entry through endocytosis (Figure 1A; Ref. 17). We found that incubating the organs at room temperature, as opposed to 37°C, decreased intracellular compartmentalization of the dye, and that 15 minutes’ incubation time was sufficient for the dye to be taken up by hair cells. We also found that loading hair cells with the maximum recommended dye concentration of 10 µM optimized fluorescence in the hair bundle. Thus, cochlear epithelia were incubated with 10 µM Fluo-4 AM for 15 minutes at room temperature, then washed three times for at least 15 minutes total to allow complete intracellular de-esterification of the dye. Most hair cells in the epithelium took up the dye and had bright Fluo-4 signal in the cell body using confocal microscopy (Figure 1B). Supporting cells took up notably less dye. Fluo-4 fluorescence was observed in hair bundles of many cells, albeit at lower intensity; saturation of the cell-body signal was necessary to detect the bundle signal (Figure 1C).

In transducing hair cells, Lumpkin and Hudspeth reported that the Ca^2+^ indicator signal using Fluo-3 was greater at the stereocilia tips, where transduction channels are located [14]. When the organ was folded in half so that hair bundles could be imaged from the side, we observed this “tip blush” of Ca^2+^ at some stereocilia tips, thought to represent Ca^2+^ entry through functional transduction channels, in 9 out of 13 cells analyzed. Bundle fluorescence was influenced by the concentration of extracellular Ca^2+^, with 0.5 mM Ca^2+^ paradoxically yielding a brighter Fluo-4 signal in the bundle compared to 1.26 mM Ca^2+^ (data not shown). Consistent with this result, high extracellular Ca^2+^ is known to partially block the Ca^2+^ current through the transduction channel [17]. We also tested Fluo-5F AM and Fluo-4FF AM, two additional dyes in the Fluo-dye family; both dyes were similarly taken up selectively by hair cells (data not shown). Because Fluo-4 has the highest Ca^2+^ affinity, cells loaded with this dye also had the brightest signal in the bundle at rest. We therefore used this dye for our experiments.

### Correlating Fluo-4 Signal with Hair Bundle Morphology

**Figure 2:**
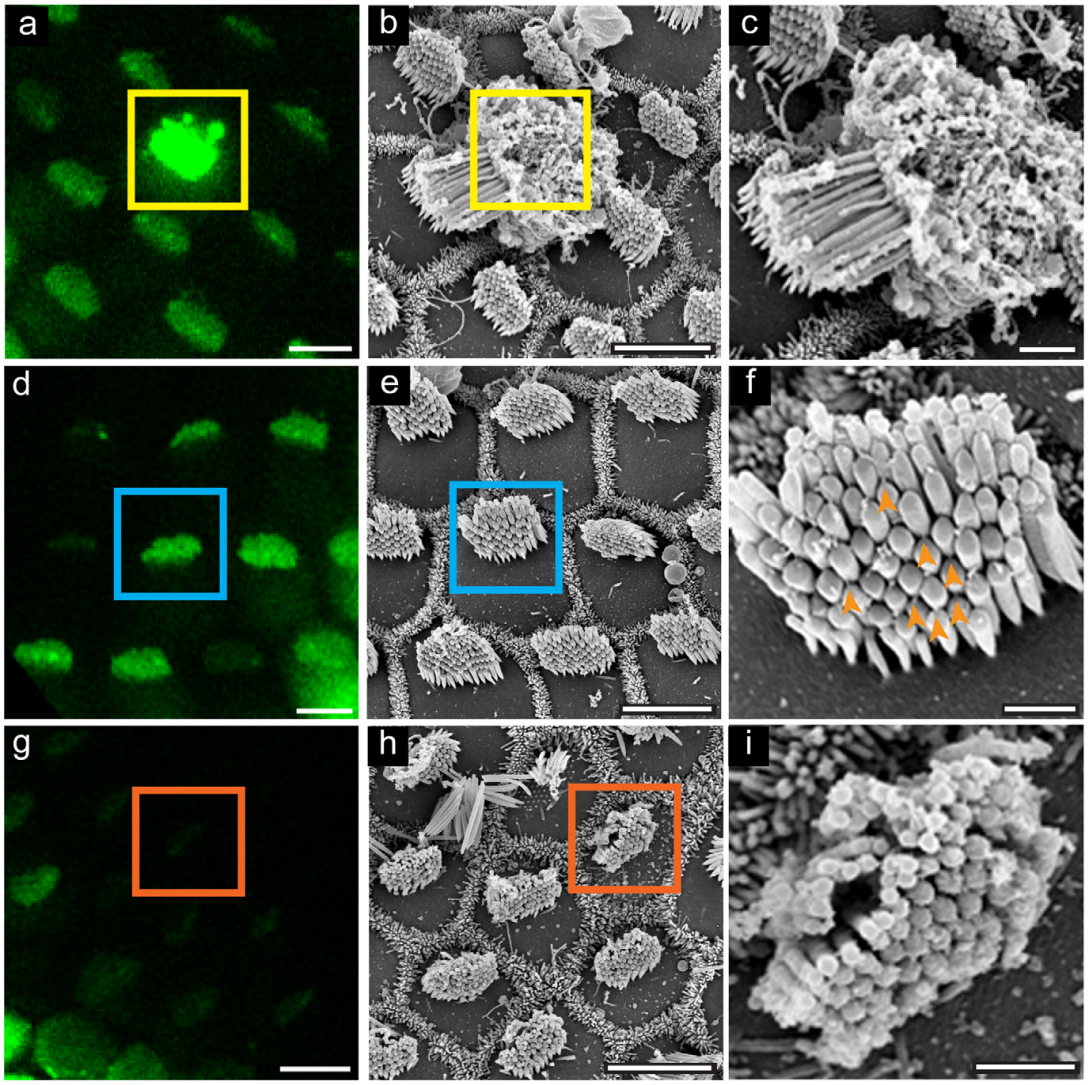
Live-cell Ca^2+^ imaging correlates with SEM ultrastructure. Organs imaged with Fluo-4 (a,d,g) were fixed and processed for SEM (b,c,e,f,h,i). (a, b, c) Cells with very bright bundle Fluo-4 represent dead or dying cells; in this example, a very bright cell became completely extruded from the ephithelium in the time between live cell imaging and fixation. (d) Hair bundles with average Fluo-4 signal have ordered stereocilia and tip links (e, f), while bundles with very low Fluo-4 signal (g) have damaged bundles (h, i). For all panels, colored boxes highlight corresponding cells in live-cell and SEM images. Orange arrowheads indicate tip links in (f). Scale bars in a, b, d, e, g and h are 5 µm, c, f, and i are 1 µm.

To examine the ultrastructure of the hair bundle and overall epithelium after Ca^2+^ imaging, organs were fixed and processed for SEM. A modified sample-preparation method that included dehydration with progressive cooling was used to preserve tip links [18]. Using landmarks observed during live-cell imaging, the same cells were located in the electron microscope. We observed three populations of hair cells, each with a distinctive Fluo-4 fluorescence in bundles that correlated with hair-cell morphology. Cells with very strong Fluo-4 signal in the bundle and cell body were undergoing extrusion from the epithelium, an indication of cell death (Figure 2A, 2B, 2C). Regions of the epithelium that had intermediate Fluo-4 signal in the bundle had organized stereocilia and tip links by SEM (Figure 2D, 2E, 2F). Bundles that had low levels of Fluo-4 during live-cell imaging were splayed and damaged, and had few tip links (Figure 2G, 2H, 2I).

**Figure 3:**
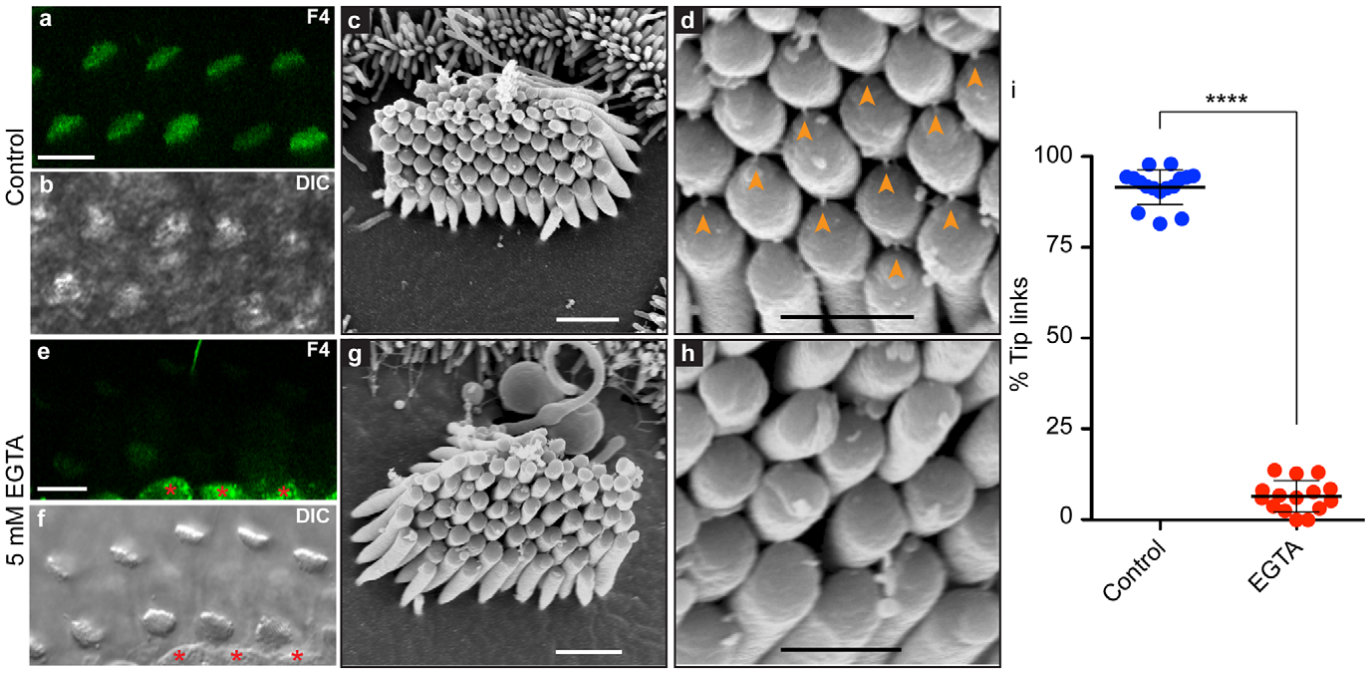
High-resolution SEM analysis of tip links. (a, b) Control hair bundles had moderate levels of bundle Fluo-4 fluorescence and many tip links at their stereocilia tips (c, d; orange arrowheads point to tip links). (e, f) Bundles treated with 5 mM EGTA for 5 min showed a marked decrease in bundle Fluo-4 fluorescence and a significant reduction in the number of tip links (g, h). Red stars in e and f highlight cell body Fluo-4 fluorescence. (i) Tip links were quantified by dividing the number of tip links in a bundle by the number of observable tip link positions (**** p<0.0001). Scale bars in a and e are 10 µm, c and g are 1 µm, and d and h are 0.5 µm.

The presence and number of tip links was further examined by high-resolution SEM. We examined regions of epithelia loaded with Fluo-4 that showed intermediate levels of bundle fluorescence; in these undamaged regions, we found tip links in 92±5% of the positions where tip links were expected (Figure 3A–D; mean ± SD, n = 16 bundles). This high density of tip links implies that the transduction complex was not damaged during dissection and suggests that Fluo-4 fluorescence in the bundle could be due to Ca^2+^ entry through functional mechanotransduction channels. Tip links are disrupted by exposure to extracellular Ca^2+^ chelators [19]. Indeed, pre-treatment of the epithelium with 5 mM EGTA lowered the Fluo-4 bundle fluorescence and significantly reduced the presence of tip links, to 7±4%, in undamaged regions of the epithelium where gross bundle morphology remained intact (Figure 3E–I; n = 15 bundles; p<0.0001).

### Direct Measurement of Changes in Fluo-4 during Mechanotransduction

**Figure 4:**
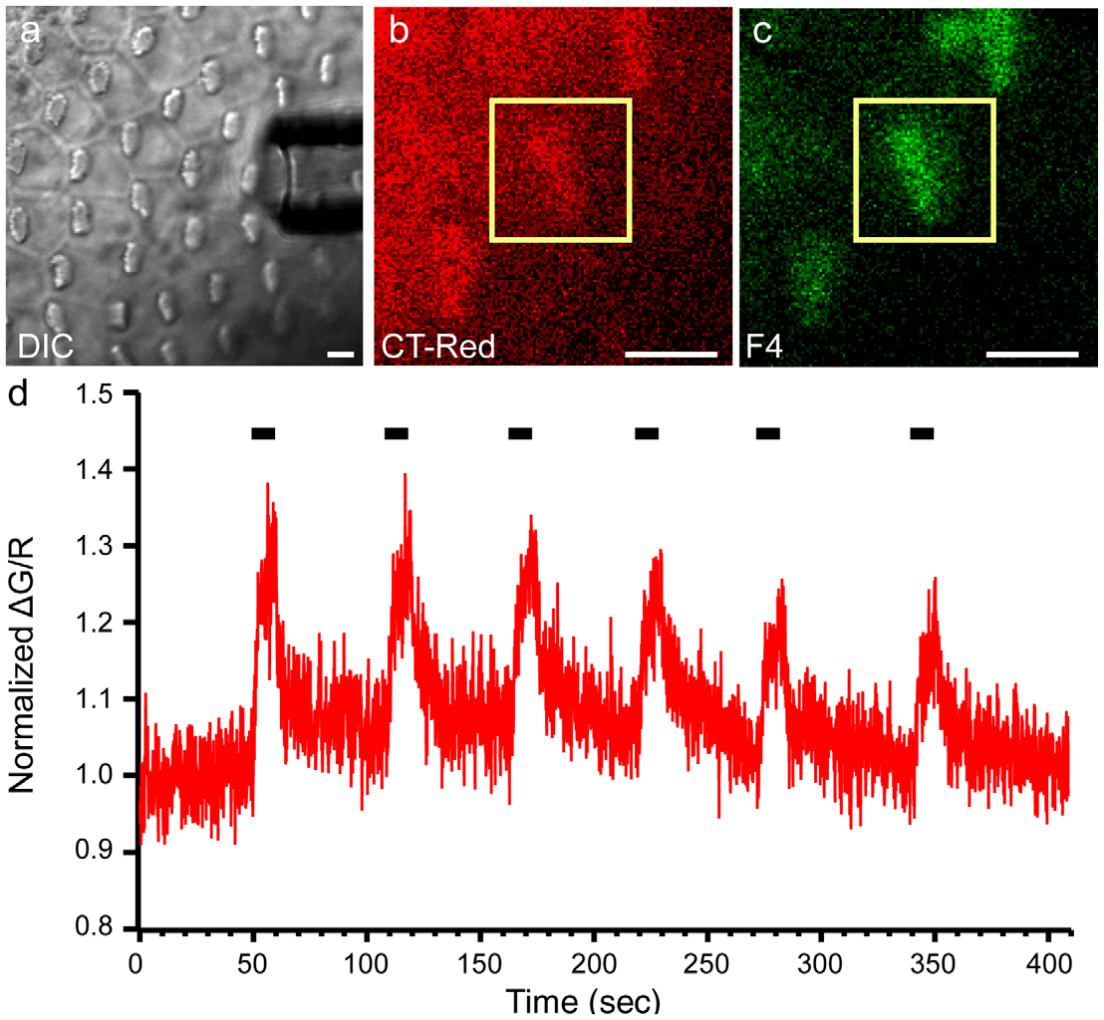
Mechanical stimulation of the hair bundle increases Fluo-4 Ca^2+^ signal. (a) A stimulating pipette was positioned parallel to the epithelium, and oriented to maximally stimulate bundles in the mid-apical region. (b) CellTracker Red (CT-Red) was used as a cell-fill to normalize the Fluo-4 bundle fluorescence (c) A single bundle region of interest (ROI; yellow box) was continually imaged. (d) Fluid jet deflection of a bundle with a 0.2 psi pulse for 10 seconds caused an increase in the bundle Fluo-4 Ca^2+^ signal, which was timed with the stimulus (black bars). Normalized ΔG/R was calculated by dividing Fluo-4 by CT-Red fluorescence, then normalizing to the average response from the first 40 seconds of recording. Scale bars in a, b, and c are 5 µm.

The two major sources of Ca^2+^ entry in the hair cell are through transduction channels in the hair bundle and voltage-gated Ca^2+^ channels in the basolateral membrane. To confirm that the Fluo-4 bundle signal reported Ca^2+^ entering through transduction channels, we mechanically activated transduction channels while simultaneously measuring Fluo-4 fluorescence in the bundle. We positioned a fluid jet stimulator parallel to the longitudinal (apical-basal) axis of the epithelium, to maximally stimulate bundles along their axis of sensitivity (Figure 4A). To normalize the Fluo-4 signal and to account for movement of the bundle, the membrane-permeable cell fill CellTracker Red (CT-Red) was loaded into the cells after Fluo-4 AM loading and wash steps; this dye was also selectively taken up by hair cells (Figure 4B, 4C). Continuous imaging of a single bundle before, during, and after mechanical stimulation of the bundle showed that normalized Fluo-4 fluorescence (ΔG/R) increased during fluid jet stimulation, with a rapid onset and slower decay (Figure 4D). Increases in Fluo-4 signal were always coincident with fluid jet stimulation. A decrease in the fluorescence maximum at stimulus onset was observed with repeated stimulations, which could represent saturation of the dye, incomplete adaptation, or breaking of tip links during fluid jet steps. These results demonstrate that mechanically stimulating the bundle with a fluid jet increases bundle Fluo-4 signal, reflecting Ca^2+^ entry into the bundle through active transduction channels.

### Quantifying Fluo-4 in Separate Intracellular Compartments

When tip links are broken, transduction channels close, which eliminates a major hair-bundle Ca^2+^ source. To quantify Fluo-4 fluorescence in response to treatments that close transduction channels, we partitioned hair-cell images into bundle and cell-body regions. We observed a punctate staining pattern in the upper half of the cell body, directly below the cuticular plate (see Figure 6). This region of the cell is an active area of endocytosis and contains a high density of mitochondria, which take up Ca^2+^ dyes [9], [20]. To avoid quantifying fluorescence due to dye trapped in intracellular compartments, we limited our cell-body fluorescence measurements to the lower half of the cell.

**Figure 5:**
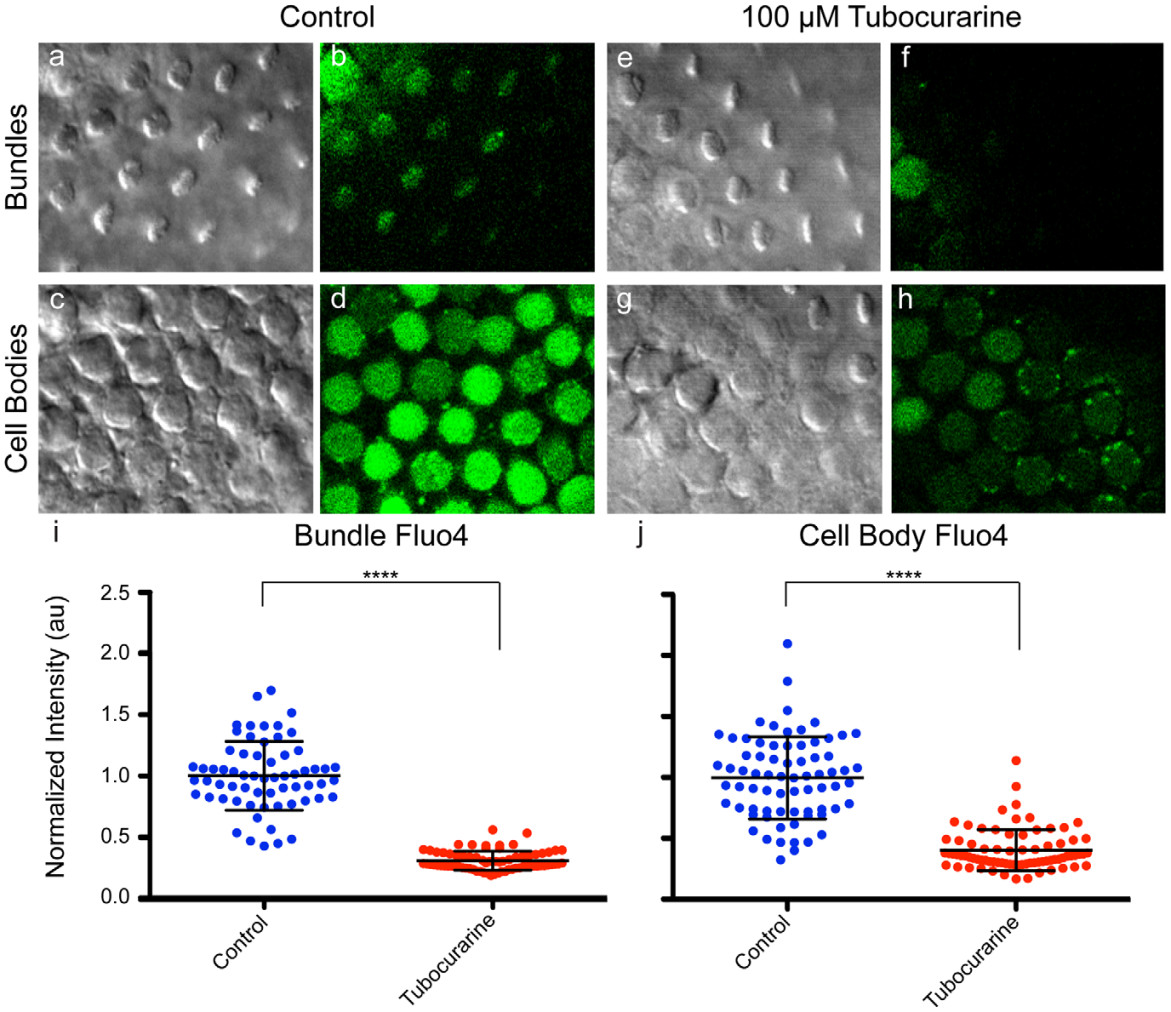
Blocking the transduction channel decreases hair-bundle and cell body Ca^2+^. (a–d) Control hair cells have Fluo-4 signal in the bundle (b) and cell body (d). (e–h) Hair cells incubated with 100 µM tubocurarine to block the transduction channel have decreased Fluo-4 signal in the bundle (f) and cell body (h). (i–j) To quantify Fluo-4 fluorescence, individual bundle and cell body ROIs were selected and integrated density of Fluo-4 channel was calculated (see Figure 6), then normalized to the control mean for each group. (a, c, e, g) Corresponding DIC images. (au = arbitrary units; control bundles n = 63, tubocurarine bundles n = 73, control cell bodies n = 72, tubocurarine cell bodies n = 77; **** p<0.0001).

We tested the effect of a transduction-channel blocker on Fluo-4 fluorescence in the hair bundle and cell body. Organs pre-treated with 100 µM tubocurarine, an open-channel transduction blocker [21], had decreased bundle and cell body Fluo-4 signal compared to untreated organs (Figure 5; control normalized bundle fluorescence = 1.00±0.28 arbitrary units (au), tubocurarine bundle fluorescence = 0.30±0.08 au, control cell body fluorescence = 1.00±0.34 au, tubocurarine cell body fluorescence = 0.41±0.17 au; normalized mean ± SD, p<0.0001 for both bundle and cell body). While the Fluo-4 fluorescence in control hair cells varied in both bundle and cell-body compartments (Figure 5), this diversity in signal likely reflects differences in the physiological state of the hair cells, as seen in Figure 2.

**Figure 6:**
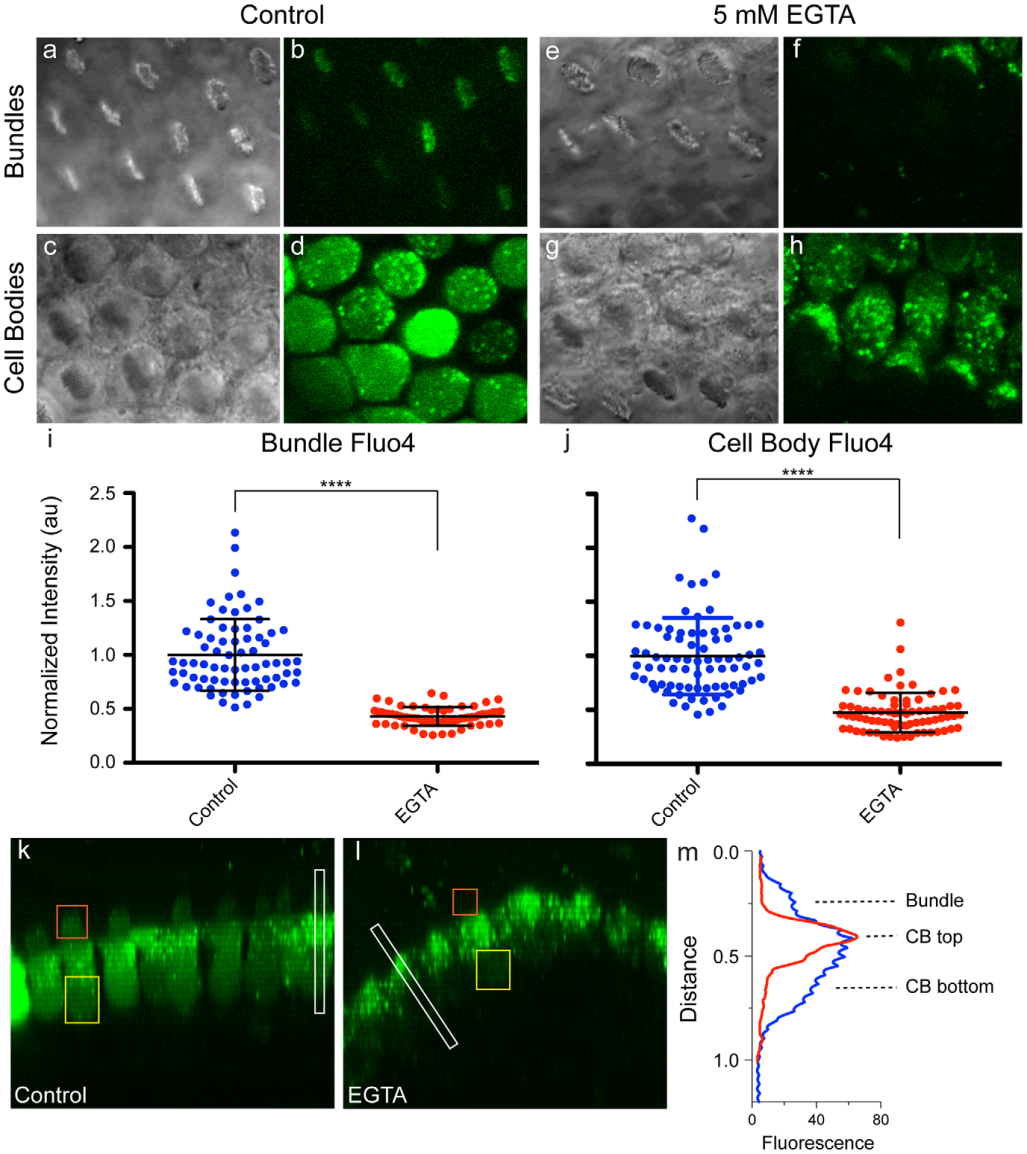
Breaking the tip links decreases hair-bundle and cell body Ca^2+^. (a–d) Control hair cells have Fluo-4 signal in the bundle (b) and cell body (d). (e–h) Hair cells pre-treated with 5 mM EGTA to break tip links have decreased Fluo-4 signal in the bundle (f) and cell body (h). (i, j) Fluo-4 fluorescence was quantified as in Figures 5 and 2. (a, c, e, g) Corresponding bundle and cell body DIC images. (AU = arbitrary units; control bundles n = 72, EGTA bundles n = 65, control cell bodies n = 79, EGTA cell bodies n = 76; **** p<0.0001). YZ-reslice images of control (k) and EGTA-treated (l) hair cells, with examples of bundle (orange square) and cell body (yellow rectangle) ROIs used for quantifying fluorescence. (m) Representative line scan profiles for the ROIs indicated by white rectangles in (k) and (l). Control hair cell (blue trace) has fluorescence in the bundle, top half of the cell body (CB top) and bottom half of the cell body (CB bottom). EGTA-treated hair cell (red trace) has fluorescence concentrated in the top half of the cell, where the dye is trapped in intracellular compartments, but lacks hair bundle and cytoplasmic CB bottom Fluo-4 signal.

Finally, to examine Fluo-4 fluorescence after tip-link breakage, cochlea were pre-treated with 5 mM EGTA for 5 minutes, then returned to normal Ca^2+^ solution for Fluo-4 AM loading and imaging. Breaking tip links significantly decreased both hair-bundle and cell-body Fluo-4 signal, from 1.00±0.33 au to 0.43±0.08 au in the bundle, and from 1.00±0.35 au to 0.48±0.18 au in the cell body (Figure 6; normalized mean ± SD, p<0.0001 for both regions). By examining the fluorescence intensity along the entire length of a cell, we further verified that Fluo-4 fluorescence in the bundle and lower half of the cell body decreased substantially after breaking tip links (Figure 6). There was no change in fluorescence in the area under the cuticular plate, where Fluo-4 dye is apparently trapped in intracellular compartments.

## Discussion

Using fluorescence arising from populations of hair cells loaded with Fluo-4 AM, we measured Ca^2+^ separately in hair bundles and cell bodies. We correlated live-cell and SEM imaging to show the relationship between Fluo-4 fluorescence and the physiological state of hair cells; bundles with moderate Fluo-4 signal at rest had ordered stereocilia and intact tip links, bundles with low Fluo-4 signal were damaged, and very brightly labeled cells were dead or dying. Direct mechanical activation of the transduction channel by fluid jet stimulation resulted in transient increases in Fluo-4 signal in the bundle; similarly, blocking the transduction channel and breaking tip links both decreased bundle and cell body Fluo-4 fluorescence, compared to untreated cells. These results show that the bundle Fluo-4 fluorescence reports Ca^2+^ entry through transduction channels, and so our method is a valuable experimental tool for investigating transduction in populations of hair cells that are relatively unperturbed.

**Figure 7:**
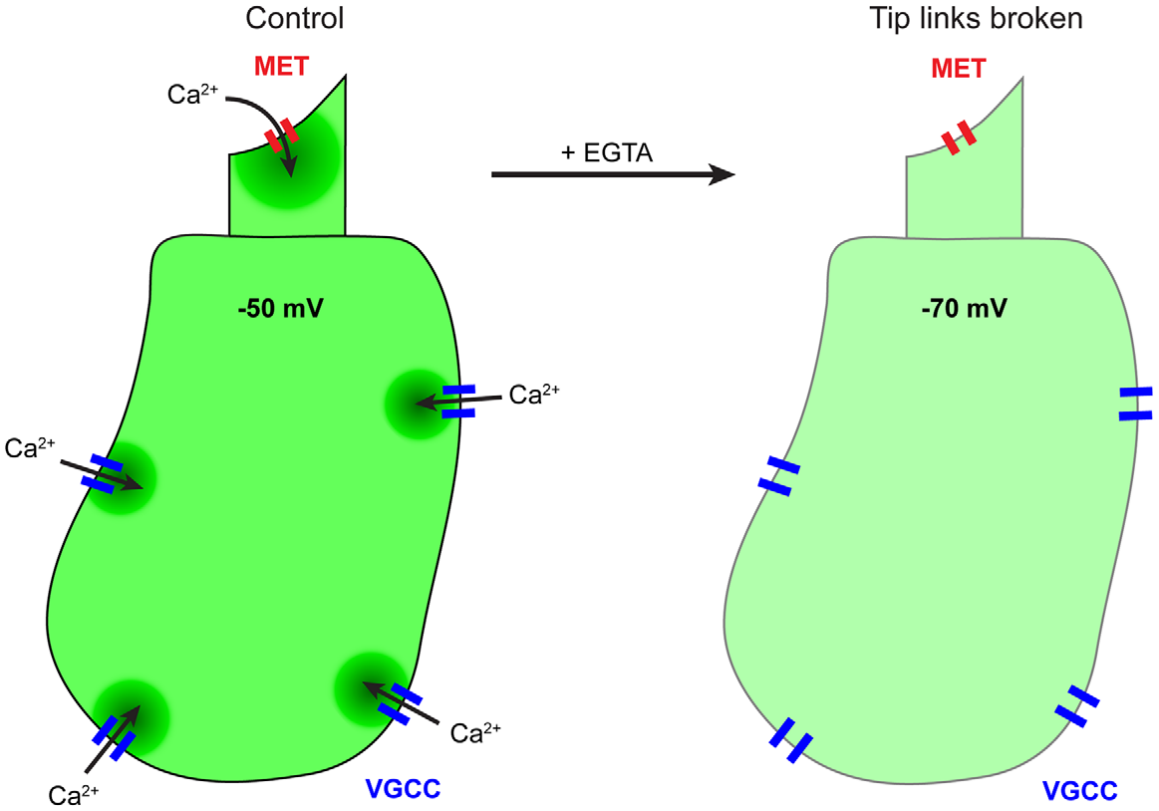
Expected physiological changes in the hair cell when tip links are broken. In control conditions, Ca^2+^ enters the hair bundle through mechanoelectrical transduction (MET) channels that are open at rest. Inward transduction current depolarizes the cell to open voltage gated Ca^2+^ channels (VGCC), which allow Ca^2+^ influx into the cell body. After breaking tip links with EGTA, the transduction channel closes, which leads to hyperpolarization of the cell and closure of voltage-gated Ca^2+^ channels, resuting in a decrease in both bundle and cell body Ca^2+^.

We observed a substantial decrease in the cell-body Fluo-4 signal upon EGTA treatment and tubocurarine block, demonstrating that activity of the transduction channel influences the concentration of Ca^2+^ in the cell body, at least under conditions of high extracellular Ca^2+^. Unlike in voltage-clamp experiments, membrane potentials of hair cells in our preparation are free to change in response to external signals. Under control conditions, tension on the gating elements of the transduction channel results in a 10–50% open probability at rest, depending on the extracellular Ca^2+^ concentration [21]–[23]. This significant open probability generates a substantial inward current that depolarizes the cell to −60 to −40 mV, which in turn should open a fraction of voltage-gated Ca^2+^ channels in the basolateral membrane [21], [23], [24] (Figure 7). Thus, the change in Ca^2+^ in the cell body in response to tip-link breakage was not unexpected; the decrease in transduction-channel conductance should hyperpolarize the cell and close voltage-gated Ca^2+^ channels.

The discovery that breaking tip links decreases both hair bundle and cell body Ca^2+^ has implications for understanding tip-link regeneration. Tip links broken by Ca^2+^ chelators regrow over ∼12 hours in culture, concomittent with the return of mechanotransduction. Moreover, addition of a Ca^2+^ ionophore, ionomycin, during regeneration blocks the regeneration of tip links, suggesting that decreased intracellular Ca^2+^ is necessary for tip-link restoration. Nevertheless, this predicted decrease in Ca^2+^ has not been observed prior to our study [5]. Based on data presented here, regeneration may require decreased hair-bundle Ca^2+^, decreased cell body Ca^2+^, or the combination of both.

Another method of assessing transduction channel activity in hair-cell populations is labeling with the lipophilic dye FM1-43, which enters the cell through the transduction channel [25]. However, multiple routes of entry for FM1-43 into hair cells have been observed, including partitioning of the dye into the outer leaflet of the plasma membrane and dye entry through P2X receptors [26], [27]. In contrast, our method of labeling hair cells with Fluo-4 AM does not rely on entry of a compound through active transduction channels, but instead provides a physiological measure of transduction-channel activity by monitoring Ca^2+^ entry into the hair bundle. While previous reports of hair-cell Ca^2+^-imaging experiments using AM dyes [10], [12], [13], [15] did not examine hair-bundle Ca^2+^ and its relationship to tip links and transduction, they did show that cell-permeable dyes like Fluo-4 AM can be used to load hair cells from various organs and species, including those from mouse and guinea pig cochlea and mouse vestibular organs. Our technique thus should be applicable to all hair-cell types.

In conclusion, this minimally invasive technique for examining intracellular Ca^2+^ in hair cells of the chicken cochlea allows us to simultaneously monitor all cells in the epithelium and to correlate Ca^2+^ signals with hair-bundle morphology, which should be useful for studying the role of Ca^2+^ in tip link regeneration. That transduction-channel activity influences both bundle and soma Ca^2+^ indicates that substantial intracellular changes occur when tip links are broken.

## Materials and Methods

### Ethics Statement

Animal experiments reported here were approved by the Oregon Health & Science University Institutional Animal Care and Use Committee (IACUC); the approval number was A684. All experiments began with euthanasia of the animal, which was carried out using methods approved by American Veterinary Medical Association Panel on Euthanasia.

### Tissue and Dye Preparation

P0 *Gallus domesticus* chickens were dissected as described previously [28], [29]; the tectorial membrane was removed with subtilisin Carlsberg (Sigma Type XXIV) protease (50 µg/ml for 15 min). Extracellular solution was used at room temperature for all dissecting, loading, and imaging steps and contained the following (in mM): 87 NaCl, 0.5 KCl, 0.5 CaCl_2_, 1.25 NaH_2_PO_4_, 2 ascorbate, 2 creatine, 6 Na-pyruvate, 75 sucrose, 25 D-glucose, 10 HEPES (pH 7.4, 310–320 mOsm). Fluo-4 AM dye was prepared as described in Figure 1, using the following reagents: Fluo-4 AM (Invitrogen, F14201), DMSO (Invitrogen, C6667), Pluronic F-127, 20% solution in DMSO (Invitrogen P3000MP). Bringing all dye reagents to room temperature before beginning was essential; moreover, all preparation and loading steps were carried out at room temperature in foil-wrapped tubes to protect dye fluorescence. To normalize for dye loading in fluid-jet experiments, the cell-permeable inert dye CellTracker Red CMTPX (CT-Red; Invitrogen C34552) was added to the organs for 15 min following Fluo4-AM loading. There was no obvious difference in the Fluo-4 signal between tall and short hair cells, and both cell types were analyzed in the group data.

### Drugs

Stock solutions of (+)-tubocurarine chloride (Sigma) at 50 mM were stored at −20°C and diluted to 100 µM in extracellular solution a few hours before use. Tubocurarine was added to organs prior to Fluo-4 AM loading and was included in all washing and imaging solutions. A stock solution of 0.5 M EGTA was stored at room temperature and diluted to 5 mM in extracellular solution lacking CaCl_2_ immediately prior to use. Organs were incubated in EGTA solution for 5 min prior to Fluo-4 AM loading, then returned to normal Ca^2+^ solution for all subsequent steps.

### Image Acquisition and Analysis

For measuring control, EGTA, and tubocurarine Fluo-4 Ca^2+^ signals, organs were imaged on an inverted Zeiss LSM710 confocal microscope (40xW/1.1 NA objective), bundles facing down. For the fluid jet experiments, an upright Olympus FV-1000 confocal microscope was used (60xW/1.0 NA or 40xW/0.8 NA dipping objectives). Dental floss attached to an electrophysiology harp was used to pin the organ down at the lagena and basal ends. Images were acquired using 1.0–2.0 µm Z-steps through the hair bundles and cell bodies. Z-stacks were processed with Imaris 3D software, where individual 2 µm XZ- or YZ-reslice images were taken through the field of hair cells. Fiji software (http://fiji.sc/wiki/index.php/Fiji) was used to select and quantify hair-bundle and cell body ROIs; integrated density was calculated and normalized to the control mean for each data set. In some experiments, organs were immediately placed into SEM fixative following imaging, fixed overnight at 4°C, and processed for SEM.

### Scanning Electron Microscopy

Organs were fixed in the dark at 4°C overnight using 2% gluteraldehyde, 1% tannic acid in 0.1 M cacodylate buffer (Electron Microscopy Sciences), washed 3 times in PBS, and stored for less than 2 days at 4°C. Organs were post-fixed in 1% osmium tetroxide (Electron Microscopy Sciences) in 0.1 M cacodylate buffer for 15 min, followed by 4–5 washes in PBS. To preserve tip links, organs were dehydrated in the following acetone series with sequential cooling, using 15 minutes for each step: 30% at 4°C, 50% at −20°C, 70% at −35°C, 90% at −35°C, 100% at −35°C, 100% at −35°C, and 100% at 4°C. Samples were kept on ice and immediately critical point dried in 100% cold ethanol. Organs were mounted in silver paint on carbon-coated stubs, sputter coated in gold/palladium to yield a ∼10 nm coat, and imaged on an FEI Sirion field-emission scanning electron microscope with 5 kV accelerating voltage.

### Fluid-jet Stimulation

Borosilicate glass pipettes with long, thin shafts were pulled and broken to yield 10–12 µm diameter tips. An open flame was used to bend the pipette along the shaft to a 30° angle, which allowed the pipette to be lowered close enough to the tissue without bumping the sidewall at the basal end of the cochlea. Pipettes were filled with extracellular solution. A Pico-Spritzer III controlled the pipette, which was mounted on a micromanipulator on the microscope stage and positioned underneath the objective. Olympus FV-1000 software was used to deliver 10 sec, 0.2 psi pulses; an in-line manometer was used to accurately measure pressure in mm Hg. By isolating a single hair bundle region of interest (ROI) of 12×20 pixels, two-color images were acquired at 70 milliseconds per frame. Data was analyzed using IgorPro software, and ΔG/R was calculated by normalizing Fluo-4 to CT-Red, then normalizing to the average response from the first 40 seconds of imaging.
